# Facial Neuralgia

**Published:** 1869-09

**Authors:** 


Facial Neuralgia.—Spasmodic or "Epileptiform."—There is a form of facial neuralgia, which, when it occurs, does so in persons past the prime of life. It is characterized by intense severity in its onset, which is also sudden It is, to some extent, hereditary. Our best treatment consists in: 1. The use of counter-irritation of a peculiar kind. 2. Nutritive tonics. 3. Subcutaneous injection of morphia, or of atropia according to circumstances. The counter irritation must not be applied to the branches of the fifth nerve, but to those of the occipital nerve. A blister at the nape of the neck is often strikingly effective in gaining a short respite. The assiduous use of cod-liver oil, or of some fatty substitute for it, should be insisted on from the first, and is of the highest consequence. (Dr. F. E. Anstie, p. 62.)

